# Learning-Based Hierarchical Decision-Making Framework for Automatic Driving in Incompletely Connected Traffic Scenarios

**DOI:** 10.3390/s24082592

**Published:** 2024-04-18

**Authors:** Fan Yang, Xueyuan Li, Qi Liu, Xiangyu Li, Zirui Li

**Affiliations:** School of Mechanical Engineering, Beijing Institute of Technology, Zhongguancun South Street, Beijing 100081, China; 3120225230@bit.edu.cn (F.Y.); 3120195257@bit.edu.cn (Q.L.); 3220220300@bit.edu.cn (X.L.); z.li@bit.edu.cn (Z.L.)

**Keywords:** graph convolutional network, decision-making algorithm, deep learning, urban autonomous driving

## Abstract

The decision-making algorithm serves as a fundamental component for advancing the level of autonomous driving. The end-to-end decision-making algorithm has a strong ability to process the original data, but it has grave uncertainty. However, other learning-based decision-making algorithms rely heavily on ideal state information and are entirely unsuitable for autonomous driving tasks in real-world scenarios with incomplete global information. Addressing this research gap, this paper proposes a stable hierarchical decision-making framework with images as the input. The first step of the framework is a model-based data encoder that converts the input image data into a fixed universal data format. Next is a state machine based on a time series Graph Convolutional Network (GCN), which is used to classify the current driving state. Finally, according to the state’s classification, the corresponding rule-based algorithm is selected for action generation. Through verification, the algorithm demonstrates the ability to perform autonomous driving tasks in different traffic scenarios without relying on global network information. Comparative experiments further confirm the effectiveness of the hierarchical framework, model-based image data encoder, and time series GCN.

## 1. Introduction

The gradual popularization of private cars in cities has provided great convenience for citizens to travel, but at the same time, it has also brought severe traffic congestion problems [1]. Normalized traffic congestion results in an exponential increase in travel time, contributing to driver fatigue and a higher risk of traffic accidents in complex traffic environments. Autonomous vehicles, equipped to handle driving tasks autonomously, offer a solution to alleviate human driving stress and enhance safety and accuracy, particularly in monotonous traffic scenarios. The decision-making layer unit is the pivotal component of the autonomous driving algorithm, equivalent to human decision-making.

Ongoing research explores various decision-making algorithms, focusing on learning-based and rule-based approaches. While rule-based algorithms [2,3,4] exhibit better stability, they reveal limitations when dealing with rich perceptual information. Decision-making algorithms based on reinforcement learning are extensively explored in autonomous driving scenarios, featuring various methods such as the DQN [5,6], DDPG [7], and AC [8]. However, due to inherent algorithmic characteristics, traditional reinforcement learning-based algorithms pose significant uncertainties in autonomous driving tasks. Consequently, some studies focus on enhancing the safety and robustness of these algorithms. Maldonado et al. analyze the impact of negative feedback on risk evaluation and decision processes in diverse driving contexts [9]. Peng et al. emphasize enhancing traffic safety with an automatic lane-change mechanism for self-driving articulated trucks [10]. They propose a novel safety lane-change path-planning and tracking-control method. Wang et al. propose a prediction method based on a fuzzy inference system (FIS) and a long short-term memory (LSTM) neural network [11]. Some studies address the issue of uncertainty by training directional models. Zhang et al. introduce a knowledge model with a problem-description layer and a problem-solving knowledge layer [12]. There are also studies based on intelligent connected vehicles for vehicle decision control [13]. While various decision-making algorithms show promising results, many are tailored to specific simplified traffic scenarios, predominantly highways [14].

The algorithm excels in simple scenarios but struggles with generalization to diverse test scenes. Its application is often confined to specific scenarios, like straight roads or single intersections, and learning-based effectiveness diminishes in complex situations. Furthermore, the algorithms rely heavily on ideal perceptual information and are tailored for V2X-enabled intelligent transportation systems. In real-world scenarios, the impracticality of seamless interconnectivity challenges algorithm performance. Hence, improving adaptability to incomplete global information is a crucial area of focus in autonomous driving research, which can be manifested in the algorithm’s ability to process raw sensor data.

End-to-end driving is a promising paradigm as it circumvents the drawbacks associated with modular systems [15]. End-to-end learning-based algorithms can theoretically cope with the type of information provided by existing mature perception systems (e.g., cameras [16], lidar [17,18], etc.). Shao et al. present ReasonNet, a novel end-to-end driving framework that extensively exploits both temporal and global information of the driving scene [19]. Hu et al. introduce a dynamic graph learning method, TAM-GCN, for the overtaking strategy, which outperforms existing methods in accuracy and safety [20]. Yang et al. further enhance decision-making in autonomous driving with the SGRL algorithm, which incorporates interactive information between agents and demonstrates superior convergence and efficiency [21]. These studies collectively highlight the potential of end-to-end learning-based algorithms in autonomous driving.

The end-to-end algorithm, which takes the image class as the input and directly outputs the vehicle’s actual action, is significantly impacted by the unpredictable nature of the model’s output. This susceptibility is a common characteristic of intricate end-to-end network structures. The fundamental challenge stems from the intricate nature of this network, seamlessly merging diverse stages of the initial process into a unified, inscrutable entity. This amalgamation transforms the model into a black box, eluding a comprehensive analysis, which introduces uncertainties about the model’s real-world performance, creating potential risks such as fatal logical errors and serious traffic accidents. As a result, the algorithm is presently confined to theoretical research, bound by its inherent mystery and the associated risks that arise when applied in real-world scenarios. The advent of convolutional neural networks (CNNs) [22] has made it feasible to directly process large-scale data such as images, point clouds, and more. With the development of dedicated image-processing networks (VGG16 [23], ResNet50 [24], and EfficientNetB7 [25]), the current target detection algorithm has been able to achieve high accuracy. Al batat et al. successfully developed an end-to-end Automated License Plate Recognition (ALPR) system utilizing YOLO for vehicle and license plate detection, achieving remarkable accuracy [26].

The modern autonomous driving system is characterized as modular tasks in sequential order, i.e., perception, prediction, and planning. In order to perform a wide diversity of tasks and achieve advanced-level intelligence, contemporary approaches either deploy standalone models for individual tasks or design a multi-task paradigm with separate heads [27]. Planning is a crucial aspect among them. Huang et al. introduced a predictive behavior-planning framework that learns to predict and evaluate from human driving data [28]. In [29], the mixture-of-experts approach is utilized to learn from human driving trajectory data to construct a multimodal motion planner. Inspired by these works and the hierarchical classification method for decision-making [30], this paper proposes a hierarchical decision-making framework. The hierarchical framework ensures superior control and stability compared to existing end-to-end algorithms. The key contributions and innovations include the following:

- This algorithm framework integrates the pre-trained target-detection model into the perceptual information preprocessing stage—the data encoder, transforming intricate image data into a state matrix conducive to decision-making networks. Adding orientation coordinates to the state matrix during construction enhances the algorithm’s adaptability to perceptual information, improving its ability to comprehend scene details.
- A state machine based on a time series GCN is introduced to align temporal concepts with the real-time dynamics of driving scenarios. The GCN outperforms the traditional CNN in capturing temporal relationships, a key enhancement that significantly boosts the algorithm’s scene-understanding capabilities.
- In contrast to the traditional end-to-end model, this algorithm adopts a hierarchical framework, rendering the entire process of perception data preprocessing, driving state classification, and action generation observable and controllable. This framework ensures enhanced stability and interpretability, unlike the opaque nature of the traditional end-to-end black-box model.
- Compared to traditional end-to-end decision algorithms, this hierarchical approach exhibits superior generalization capabilities across various application scenarios, meeting the demands of autonomous driving tasks for predetermined trajectories on standardized roads.

The paper unfolds as follows: Section 2 provides a detailed introduction to the proposed hierarchical decision-making framework. Section 3 introduces the implementation of the experiment. Section 4 summarizes and compares the algorithm’s experimental results and evaluation indexes in the autonomous driving task. Finally, Section 5 derives the conclusion and proposes future improvement directions.

## 2. Methods

**Figure 1:**
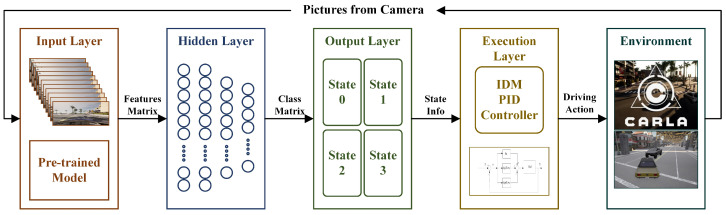
Hierarchical algorithm framework diagram. (The feature matrix comprises the feature vectors of the unified format obtained by the model preprocessing and coding of the image. The Class Matrix represents the representation matrix of the neural network classification results—the state machine. State info represents several driving states the vehicle needs to divide according to the predetermined route in the urban scene, corresponding to different control parameters of the next layer. Driving action is the scale-calibrated information such as steering wheel angle, throttle, and brake, which can be directly executed by the vehicle generated by the PID controller based on the predetermined driving route).

The hierarchical framework presented in this paper is structured into four layers, functioning sequentially in the information flow essential for vehicle autonomous driving. The depiction of this hierarchical framework is presented in Figure 1.

### 2.1. Input Layer—Image Preprocessing and Encoder

In the context of human drivers, the eyes serve as the primary source of environmental information. Hence, it is commonplace to equip autonomous vehicles with cameras. Images offer a distinct advantage regarding the abundance and richness of information, providing a comprehensive view of the driving environment. To ensure an ample supply of information, the algorithm captures camera data from four directions around the vehicle. This framework breaks the algorithm’s dependency on global network information by using an array of images as the input for determining the vehicle’s driving state.

#### 2.1.1. Model-Based Image Preprocessing

Traditional algorithms employing image information as the input often utilize convolutional neural networks (CNNs) as the processors. While these algorithms have demonstrated success in image recognition, their direct application in autonomous driving is primarily in the end-to-end form, posing challenges related to convergence and high training costs.

Some existing image-recognition algorithms can extract all target types and their occupied pixel panes in the image, corresponding to the visual information preprocessing in autonomous driving. YOLO [31] is a mature multi-target-recognition algorithm that offers several pre-trained models. The latest version of YOLO v9 [32] is currently the new SOTA for target detection. Utilizing this model for image preprocessing allows for the direct extraction of pertinent targets in the image and their position information in the field of view.

#### 2.1.2. Encoder and State Matrix Construction

The image data processed by YOLO are transformed into a series of state information distinct from the original pixel points. This information encompasses the identified target type, the pixel position of the target in the image, and the confidence information associated with the identification. Additionally, based on the input image number, it becomes possible to differentiate the camera information corresponding to each target. This information, in turn, signifies the directional position of the target relative to the vehicle. To facilitate the subsequent network’s input process, it is imperative to establish standardized coding rules for generating a state matrix.

**Figure 2:**
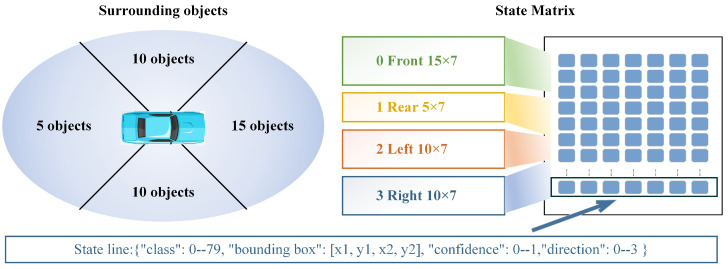
State matrix coding rule diagram. (Considering that the influence of the environment in front of the vehicle on driving behavior is much stronger than that in the rear, the five target quotas in the rear are allocated to the front. Each line in the dataset represents a discerned target, encapsulating vital information across seven dimensions. These dimensions encompass the object’s categorical assignment within the range of 0 to 79, the precise corner coordinates outlining the bounding box, the confidence level associated with the identification, and the directional code signifying the object’s position in relation to the vehicle, denoted by values ranging from 0 to 3).

The specification of the state matrix hinges on the effective number of targets identified in the images captured from all directions. In the *Carla* simulation platform, 100 autonomous NPC vehicles and 50 NPC pedestrians are introduced into the town map, which has a side length of 500 m, thus simulating high-density traffic scenes. By tallying the number of targets identified by the four cameras during the agent vehicle’s drive over a simulation time of 5 h, a total of 18,000 frames of data are obtained. The average number of targets in the image is calculated to be 10.107. Consequently, the specification of the state matrix can be configured as depicted in Figure 2.

### 2.2. Hidden Layer—Network Structure

**Figure 3:**
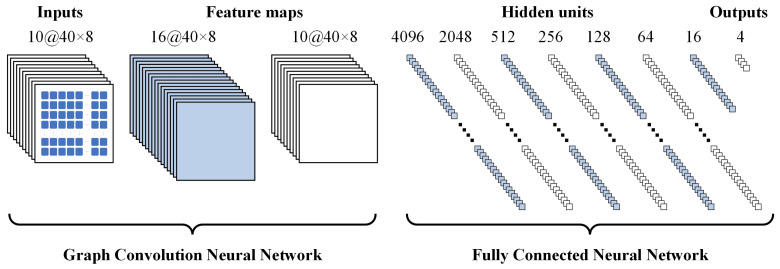
Neural network hidden layer diagram.

The feature matrix obtained by preprocessing has been significantly simplified regarding the dimensions and specifications for the original image data. However, it still contains much information and is unsuitable for direct driving state determination. Recognizing the outstanding capabilities of neural networks in handling large-scale information, this algorithm leverages them to analyze the state matrix. Since vehicle driving is continuous, historical information becomes crucial in current decision-making. Hence, the algorithm innovatively introduces a GCN based on time series information. The overall structure of the neural network hidden layer is depicted in Figure 3.

#### 2.2.1. Time Series GCN

Time series data inherently contain temporal dependencies, meaning that the value of a variable at one-time point is often dependent on its previous values, which is necessary for the driving state determination. GCNs can effectively capture these dependencies by incorporating information from neighboring time points in the graph structure, allowing them to model temporal relationships more effectively than traditional neural networks.

**Figure 4:**
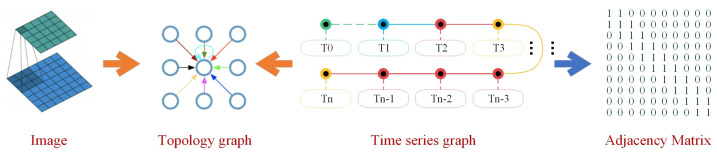
Time series graph convolution equivalent diagram. (As part of the initial data processing, YOLO is employed to extract fundamental information from image data, forming the basic nodes of the topological graph. The interconnected relationships within the topological graph are then sequentially established through the timeline in this paper. Each timestamp’s image, along with the extracted feature information, collectively shapes the nodes of the GCN. Simultaneously, the timeline serves as the edges of the GCN, playing a crucial role in determining the content of the adjacency matrix).

GCNs can directly operate on graphs and utilize their structural information. This concept is transplanted from the image field to the graph field. However, images typically exhibit a fixed structure, whereas the structure of a graph is more flexible and intricate. The fundamental idea behind GCNs is to consider all neighboring nodes and the feature information embedded in each node. This approach enables convolutional calculations on the topology graph. If the information is treated as a node at each moment, the time series information essentially forms a distinct topology, as illustrated in Figure 4.

In the GCN section, each time series’s state matrix is treated as an individual node, and the temporal relationships can be likened to the configuration of an adjacency matrix. (In the model proposed in this paper, the adjacency matrix is a 10-dimensional square matrix with a diagonal and sub-diagonal of 1, as shown in Figure 4. This implies that the current time and the initial nine timestamps carry equal significance, with interactions occurring solely between adjacent timestamps). Through graph convolution, the state matrix, when coupled with timing information, can be effectively processed. This approach proves more focused than a regular fully connected neural network when analyzing the sequential relationships among states. A 10-16-10 graph convolutional structure was chosen for the graph neural network segment.

#### 2.2.2. Fully Connected Neural Network

The feature matrix computed by the GCN requires a detection head for the final classification output, which can be achieved using a fully connected neural network. There is no rigid standard for the depth of the network and the number of nodes in each layer. Consequently, the ultimate network structure must be determined through a series of comparative experiments. Considering that the number of output features in the GCN section is 10×40×8=3200, a control group can be established as outlined in Table 1 based on empirical considerations.

**Figure 5:**
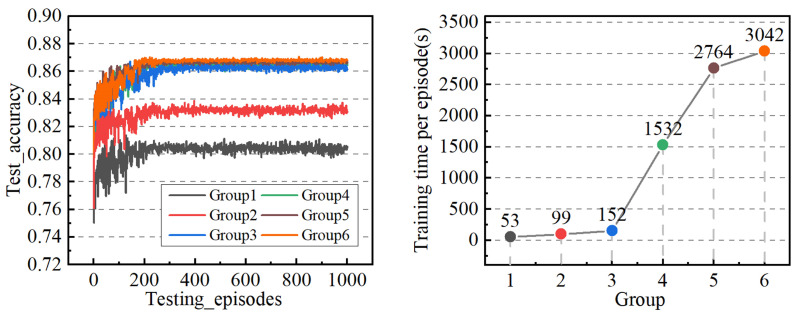
Schematic diagram of the experimental results of the comparison of the network structure. (The data analysis reveals a notable enhancement in accuracy for Groups 3 to 6. Additionally, there is a substantial reduction in training costs observed for Groups 4 to 6).

The groups mentioned above undergo separate training, with the total length of the training dataset amounting to 81,268. Following each iteration of traversing the dataset, the model’s prediction accuracy is evaluated using the testing dataset comprising 19,068 instances. The experimental results are illustrated in Figure 5. Notably, starting from Group 3, there is a significant increase in the model’s prediction accuracy, albeit accompanied by a gradual rise in the time required for a single training episode. Subsequent experiments (Groups 4 to 6) exhibit spikes in the computing time due to the deepening of the networks, but the corresponding improvements in accuracy are not as pronounced. Therefore, it is deemed more appropriate to preliminarily determine the network structure parameters based on the configuration of Group 3, which has been utilized in the proposed framework outlined in this paper.

### 2.3. Output Layer—Determination of State Machine

The vehicle typically follows a predetermined route by default throughout the driving process to ensure it reaches its destination. However, in actual driving scenarios, occasional disturbances arise due to interactions with the external environment. The classification of a driving state machine can characterize these disturbances. Drawing inspiration from the driving behavior of human drivers on urban roads, vehicle driving states can be classified as follows. This classification also determines the dimension (four) of the model output layer:

1. Conventional driving state: The vehicle is driving on a conventional road section without traffic signal restrictions and maintains a safe distance from surrounding vehicles.
2. Car-following state: In a scene with a large traffic flow, the vehicle needs to follow the front vehicle and keep a fixed safe distance.
3. Traffic intersection driving state: There are interactive behaviors generated by reverse and vertical vehicles in the future driving area of the target vehicle, which includes all intersection types such as crossroads, T-junctions, and roundabouts, with traffic signal lights.
4. Emergency braking state: The vehicle performs emergency braking before the imminent collision and the violation of traffic rules such as running a red light.

The classification of driving states utilized in our study has been carefully considered to simultaneously meet the requirements of the comprehensive coverage of urban driving tasks and distinct differentiation of the control parameters. Any driving task in urban settings can be entirely composed of these four states.

### 2.4. Execution Layer—Rule-Based Vehicle Motion Control

Rule-based vehicle motion control essentially constitutes a path-tracking algorithm that considers various driving states. The end-to-end learning-based autonomous driving algorithm exhibits serious instability. For the subsequent verification experiment, this paper opts for a rule-based algorithm to execute the vehicle’s specific driving actions (throttle, steering, braking, etc.). Considering the characteristics of the rule-based algorithm, its control logic must be designed distinctively for various driving states. By simplifying the driving task into four predefined driving states for the output, the previous algorithm facilitates this aspect significantly.

#### 2.4.1. PID-Based IDM Control Algorithm

**Figure 6:**
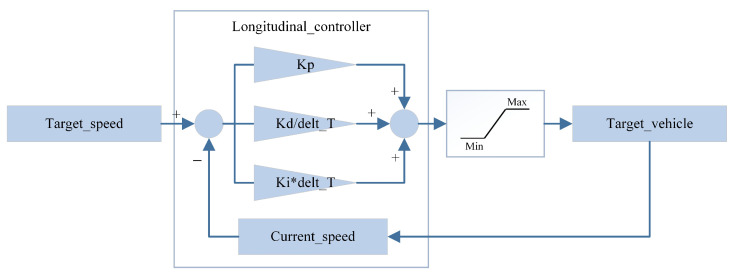
Longitudinal PID control algorithm.

**Figure 7:**
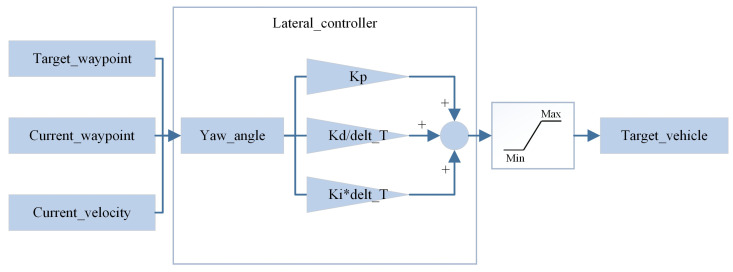
Lateral PID control algorithm.

The principle of the PID-based IDM control algorithm mentioned above is illustrated below. The PID control process is divided into two components: lateral control and longitudinal control and generating the steering wheel angle and throttle/brake control signals, respectively, as depicted in Figure 6 and Figure 7.

During the experiment, adjusting the parameters of the PID controller is a primary task. Adjusting the PID parameters is an empirical process typically involving experimentation and observing the system’s response. After the first set of PID parameters is stabilized and the agent is controlled to accelerate beyond 50 km/h, it became evident that the control effectiveness was notably compromised. In the case of the speed exceeding 50 km/h, the PID parameters are re-calibrated. The adjustment ensures stable operation in the speed range of 50 to 100 km/h, and the upper limit setting refers to the maximum allowable speed of the Beijing Expressway. Recognizing the direct correlation between vehicle control stability and speed, this paper designates the speed of 50 km/h as the critical point and establishes two different sets of PID parameter configurations, as outlined in Table 2.

#### 2.4.2. Conventional Driving State

- Target speed: 30 km/h.
- Throttle control: as shown in Algorithm 1.
- Steering control: as shown in Algorithm 2.

**Algorithm 1.** Acceleration Control of the Conventional Driving State
1. control=carla.VehicleControl().2. Target_speed=get_target_speed()3. $\mathrm{Acceleration}\_\mathrm{value}\;=\;\mathrm{Longitudinal}\_\mathrm{PID}\_\mathrm{controller}\left(\mathrm{Target}\_\mathrm{speed}\right)$4. if Acceleration_value≥0:

control.throttle=Acceleration_value




control.brake=0

5. else

control.throttle=0




control.brake=abs(Acceleration_value)



**Algorithm 2.** Steering Control of the Conventional Driving State
1. control=carla.VehicleControl()2. Way_point=get_way_point()3. $\mathrm{Steering}\_\mathrm{value}=\mathrm{Lateral}\_\mathrm{PID}\_\mathrm{controller}\left(\mathrm{Way}\_\mathrm{point}\right)$4. control.steer=Steering_value

#### 2.4.3. Car-Following State

- Target speed: as shown in Algorithm 3.
- Throttle control: The throttle and steering wheel angle calculation in this state is completely consistent with that of the conventional driving state, except for the target speed.

**Algorithm 3.** Target Speed of Car-Following State
1. Target_vehicle=get_target_vehicle()2. Δv=abs(self.speed-get_speed(Target_vehicle))3. Time_to_collision=distance(self.vehicle,Target_vehicle)/Δv4. if 0Time_to_collisionsafety_time:

Target_speed=min(get_speed(Target_vehicle)-5,min(self.max_speed,25))

5. else if safety_timeTime_to_collision2*safety_time:

Target_speed=min(max(self.min_speed,get_speed(Target_vehicle)),



min(self.max_speed,25))

6. else

Target_speed=min(self.max_speed,25)



#### 2.4.4. Traffic Intersection Driving State

When the vehicle is navigating a traffic intersection environment, there is a higher likelihood of overlapping with the anticipated trajectories of multiple vehicles compared to a conventional environment. Hence, it becomes necessary to reduce the speed. The control algorithm in this mode mirrors the conventional driving state, with the only difference being a reduction in the target speed by 5 km/h.

#### 2.4.5. Emergency Braking State

- Target speed: 0 km/h.
- Throttle control: 0 (the throttle control is set to 0).
- Brake control: 1 (the brake control quantity is set to the maximum).

## 3. Experiment

### 3.1. Simulation Environment Set Up

The utilization of simulation scenarios serves two primary purposes: data collection and model verification. The simulation scene is consistently set by selecting *Carla*’s built-in Town series map, and NPC vehicles and pedestrians are generated randomly within this environment. The agent vehicle is initially placed at a randomly chosen generation point, and the control mode for the agent vehicle (whether it utilizes a built-in algorithm or a trained model) can be selected based on the specific experiment requirements.

This standardized approach ensures consistency across experiments and allows for efficient comparison of the results. The *Carla* Town series map provides a realistic and diverse environment for testing, while the random generation of NPCs introduces variability to the scenarios. Researchers can then choose between the built-in algorithm or a trained model to control the agent vehicle, tailoring the experiment to their specific needs. This methodology provides a robust foundation for both data collection and model verification, contributing to the reliability and repeatability of simulation-based experiments.

### 3.2. Self-Built Dataset

**Figure 8:**
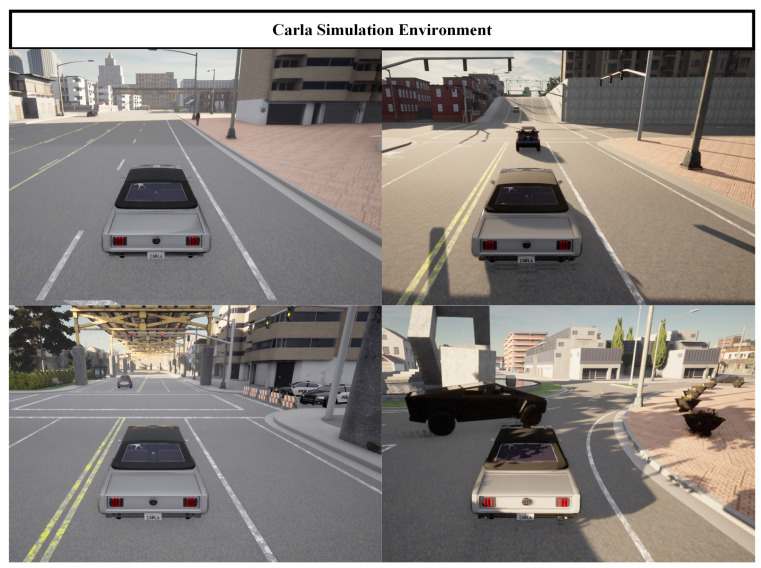
*Carla* simulation scenario diagram.

The learning-based algorithm model demands an extensive dataset for training, necessitating data collection through experiments. *Carla*, a well-established urban traffic scene simulator, can generate highly realistic urban maps within its environment. Moreover, it allows the definition of vehicle and pedestrian NPCs for interactive purposes. The ultimate simulation environment is illustrated in Figure 8.

**Figure 9:**
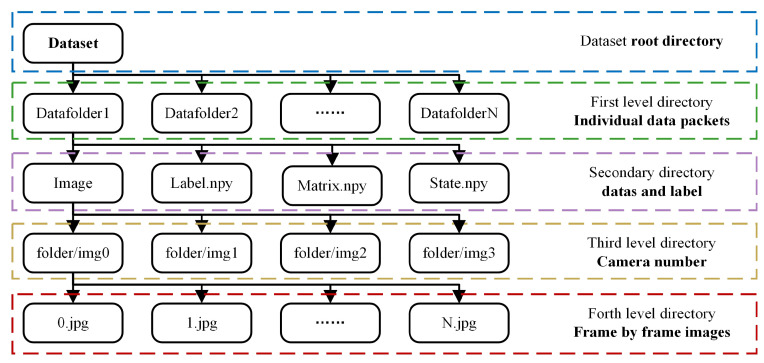
Dataset directory structure diagram.

The vehicle’s driving state information (label value) can be directly obtained in the simulation environment. By allowing agent vehicles to circulate randomly on various routes within the city, a substantial number of images, state matrices, and driving state (label value) information can be collected to create a comprehensive dataset. The driving states are directly derived from parameter queries within the *Carla* simulator, resulting in four distinct categories. This serves as the basis for the aforementioned state classification. To ensure comprehensive data coverage, during the data-collection process, factors such as the time of day and lighting conditions in the simulation environment vary randomly, as illustrated in Figure 8. The final dataset has a length of 396,058, and the composition of the dataset is depicted in Figure 9.

### 3.3. GCN Model’s Training

The comparative experiment was devised to validate the effectiveness of the proposed algorithm’s innovation in the model-training segment. The detailed design of the training program is outlined in Table 3.

The data-flattening process and fully connected network (FCN) processing are standard operations in deep learning and were consequently chosen as the baseline group. For image preprocessing, VGG16 [23], ResNet50 [24], and EfficientNetB7 [25] were selected as the control group, given the robust capabilities in image recognition. Regarding processing timing information, LSTM [33] was chosen as the control group, recognized for its proficiency in handling sequence information.

In the model-training section, the following pre-configuration measures were implemented:

1. In the dataset loading process, considering a large amount of information in the image data, the ***Dataloader in Pytorch*** is established by reading the file list.
2. To improve the training efficiency, ***ReduceLROnPlateau*** and ***Adaptive Gradient*** are introduced into the model training.
3. Considering the imbalance of the proportion of different tags in the database, the data are uniformly and randomly sampled according to the number of different tags in the dataset-reading process. The final training set length is 81,268, the test set length 19,068, and the total amount of data more than 100,000.

### 3.4. Co-Simulation Verification

**Figure 10:**
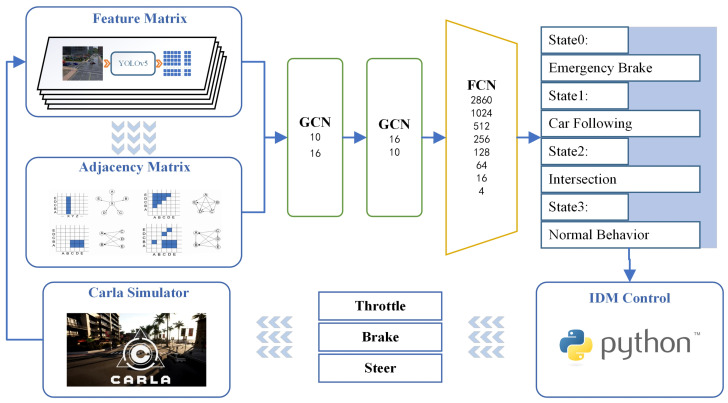
Co-simulation verification process diagram (the detailed descriptions of the feature matrix and the adjacency matrix can be found in Section 2, “Input Layer—Image Information and Preprocessing”, in the preceding section. GCN: Graph Convolution Neural Network; FCN: Fully Connected Neural Network; IDM: Intelligent Driver Model).

In the co-simulation verification, the agent vehicle was configured to operate as an autonomous vehicle within a realistic city scene. The information available in the simulation environment was restricted to the vehicle’s location, speed, onboard sensor (camera) data, and the predetermined driving route. These parameters align entirely with the characteristics of the current real traffic scene. The overall process is illustrated in Figure 10.

Two control groups were set up in this experiment:

1. Baseline group: The agent vehicle can obtain all global information from the simulation environment, including the map location, vehicle location, vehicle speed, and traffic status.
2. Hierarchical decision-making framework group: The agent vehicle can only obtain its vehicle speed information and the image information of the top four cameras and input the above information into the pre-trained optimal model to obtain the vehicle’s state information to complete the control of the vehicle.

The final indicators were evaluated as follows.

1. Average vehicle speed: The efficiency of the vehicle in completing the driving task has always been an important indicator for evaluating the autonomous driving algorithm.
2. The number of collisions: The vehicle’s driving safety is the bottom line of the application of the automatic driving control algorithm.
3. Destination arrival rate: The automatic driving of vehicles to reach the predetermined target point is the completion of a task, and the completion of the task of the vehicle is also a critical assessment index for the automatic driving of vehicles.
4. Emergency braking frequency: The effective emergency braking of the vehicle can avoid the occurrence of traffic accidents, but frequent braking will lead to the decline of member comfort and the improvement of vehicle energy consumption.
5. Control cycle: The shorter the control cycle, the higher the computational efficiency of the representative algorithm, which can adapt to improving driving requirements such as higher vehicle speed.

## 4. Result

### 4.1. GCN Model’s Training Result

**Figure 11:**
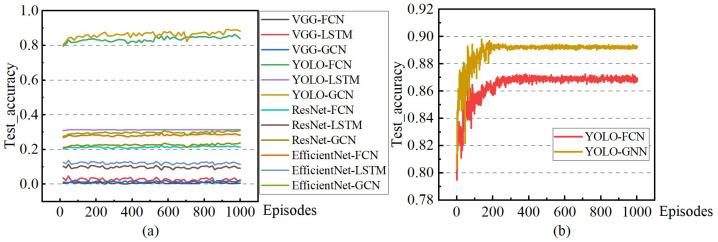
Schematic diagram of model prediction accuracy change (**a**): Changes in model prediction accuracy during the training process. (**b**): For the convenience of comparison, groups involving YOLO are presented separately. The data clearly indicate that the YOLO model integrated with GCN exhibits superior comprehensive performance among the evaluated models.

**Figure 12:**
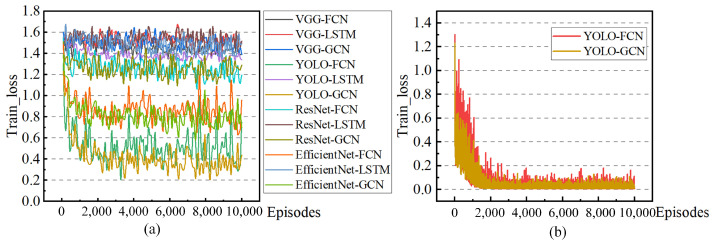
Schematic diagram of loss change in the training process (**a**): Changes in model loss during the training process. (**b**): For the convenience of comparison, groups involving YOLO are presented separately. The data clearly indicate that the YOLO model integrated with GCN exhibits superior comprehensive performance among the evaluated models.

The primary evaluation metrics for the model training process are the real-time prediction accuracy and the loss value during training. The trends of these two indicators are visualized in Figure 11 and Figure 12. (Accuracy represents the recognition accuracy of the model for the current driving state label. The loss represents the default loss calculation in the deep learning process, which is used to characterize the convergence direction and effect of model training. The episode is an inherent aspect of the model training process, representing a cycle of training and testing. This paper symbolizes the entire journey from generating the agent vehicle to completing a specific task—arriving at the destination).

The comprehensive comparison diagram in Figure 11a indicates that the two experimental groups, YOLO-FCN and YOLO-GCN, exhibit notable advantages over the other four groups. This paper conducts a detailed comparative analysis of the two groups in Figure 11b. Examining the data trends, it is evident that both groups achieve a satisfactory convergence effect. Regarding the convergence value, YOLO-GCN outperforms YOLO-FCN, improving prediction accuracy by 2%. The outcomes of the comparative experiments affirm that the YOLO preprocessing and the proposed time series GCN effectively enhance state prediction in this experimental scenario.

The comprehensive comparison diagram in Figure 12a substantiates that both YOLO-GCN and YOLO-FCN exhibit significant improvements in the convergence of model training. The primary reason lies in the effectiveness of YOLO preprocessing in reducing the complexity and uncertainty of input information, thereby making model training more focused and directional. The loss trends of YOLO-GCN and YOLO-FCN depicted in Figure 12b are stable and convergent, confirming the normality and effectiveness of the model training for both groups.

The results in Figure 11 and Figure 12 mutually reinforce each other, demonstrating that the enhanced model prediction accuracy brought about by the newly proposed YOLO preprocessing and the concept of time series GCN stems from an improvement in the practical convergence ability of the model training process.

### 4.2. Co-Simulation Verification Result

**Figure 13:**
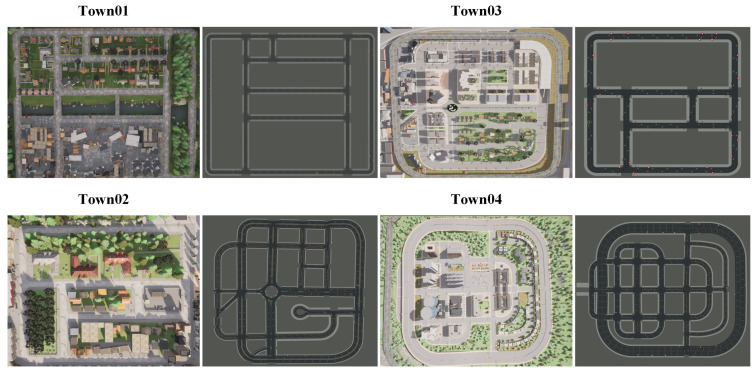
Scenario maps for verification in *Carla*. (Town01: A simple town with a river and several bridges. Town02: A simple town with a mix of residential and commercial buildings. Town03: A larger city map with roundabouts and large intersections. Town04: A square grid town, with intersections and a bridge. There are multiple lanes in each direction. It is useful for performing lane changes).

The *Carla* simulation environment offers ten optional maps (Town01 to Town10). Four scenarios with traffic lights were chosen for the co-simulation verification, as illustrated in Figure 13.

In the four *Carla* simulation scenarios, 60 NPC vehicles and 40 NPC pedestrians were randomly introduced. These NPCs can autonomously navigate using *Carla*’s built-in traffic controller. In each experiment episode, the agent vehicle is randomly positioned at a starting point, and a destination location is randomly selected for driving. The episode concludes in the event of a collision. For the two experimental control groups, 1000 episodes were conducted on each map. The comparative results are presented in Table 4. The YOLO-GCN algorithm model, exhibiting the best performance in the preceding section, was employed in the hierarchical decision-making framework group—this paper.

The distinct variations in the road traffic environment, lighting conditions, and interactive objects (such as pedestrians and cars) across the four map scenes provide a robust foundation for evaluating the model’s generalization ability index. These diverse scenarios are essential for thoroughly testing the model’s adaptability across different real-world settings. The Baseline group utilizes the rule-based autonomous driving algorithm embedded in the *Carla* environment, enabling direct access to all information within the environment. Consequently, it stands as a fully idealized control group with distinct advantages. The comparison between the model proposed in this paper and the Baseline group is a comprehensive validation, providing a thorough assessment of the proposed model’s effectiveness. Within the *Carla* environment, the agent vehicles perform actions solely generated by the algorithm, without any additional driving assistance. This set up mirrors the control methods of human drivers in the real world. The configurations for the validation experiments were designed to faithfully replicate actual autonomous driving situations.

The results strongly indicate that the vehicle control performance obtained by the model proposed in this paper, relying on visual data, is largely comparable to the idealized Baseline group. This finding serves as substantial evidence to validate the effectiveness and generalization capability of the proposed model. It can be concluded that the algorithm framework proposed in this paper demonstrates similarity to the Baseline group across all evaluation indicators, with a maximum difference of 7.56%. This substantiates that the algorithm framework presented in this paper effectively ensures the successful completion of the vehicle’s autonomous driving task in a traffic scenario with incomplete global information. Additionally, the algorithm framework’s performance on relevant indicators supports its seamless integration as the state prediction module for autonomous vehicles. Moreover, the table indicates that the newly proposed hierarchical framework can adeptly handle autonomous driving tasks in four distinct maps, showcasing the algorithm’s excellent generalization ability.

## 5. Conclusions

This paper introduces a hierarchical framework for autonomous vehicle driving tasks in non-networked scenarios, which can execute autonomous driving tasks in different environments without relying on global network information. Given the safety constraints of real vehicle experiments, validation experiments based on *Carla*, which can fully simulate real-world scenarios, represent the most convincing simulation approach currently available. The comparative experiment section validated the effectiveness of each of these innovations individually. The YOLO pre-training model achieved nearly a 90% recognition accuracy for vehicle driving states under identical training conditions. This level of accuracy surpasses what can be achieved by the basic FCN and the well-established VGG16, ResNet50, and EfficientNetB7. Incorporating the time series GCN network resulted in a 2% enhancement in recognition accuracy compared to the non-sequential FCN structure. Additionally, it outperformed the LSTM algorithm significantly in terms of processing timing information.

Indeed, with the current model achieving a prediction accuracy of around 90%, future work could explore the application of additional image preprocessing models to further enhance the prediction accuracy and overall performance of the autonomous driving algorithm. Investing in a more robust training hardware platform and allocating ample model training time can contribute to achieving superior training results. Additionally, incorporating actual vehicle experiments could provide a more thorough validation of the algorithm’s effectiveness in real-world scenarios. To directly validate the model algorithm, actual vehicle experiments can be arranged for direct verification once the security of the future algorithm is further improved.

## Figures and Tables

**Table 1:** Network structure control experiment settings

Group Number	Fully Connected Layer Structure Setting
01	2048-512-128-32-8-4
02	2048-2048-512-128-32-8-4
03	4096-2048-512-128-32-8-4
04	4096-4096-2048-512-128-32-8-4
05	2048-1024-512-256-128-64-32-8-4
06	4096-2048-1024-512-256-128-64-32-8-4

**Table 2:** PID parameter setting.

Speed	ΔT	Direction	$K_{p}$	$K_{d}$	$K_{i}$
0–50 km/h	0.05 s	Lateral	0.58	0.02	0.5
		Longitudinal	0.15	0.05	0.07
50–100 km/h	0.05 s	Lateral	0.75	0.02	0.4
		Longitudinal	0.37	0.024	0.032

**Table 3:** Comparative experiment settings.

Training Group	Image Processing	Timing Series Processing
**Comparison 1**	VGG16	Flatten and FCN
**Comparison 2**	VGG16	LSTM
**Comparison 3**	VGG16	Timing-based GCN
**Comparison 4**	ResNet50	Flatten and FCN
**Comparison 5**	ResNet50	LSTM
**Comparison 6**	ResNet50	Timing-based GCN
**Comparison 7**	EfficientNetB7	Flatten and FCN
**Comparison 8**	EfficientNetB7	LSTM
**Comparison 9**	EfficientNetB7	Timing-based GCN
**Comparison 10**	YOLO	Flatten and FCN
**Comparison 11**	YOLO	LSTM
**Proposed algorithm**	YOLO	Timing-based GCN

**Table 4:** Experimental results of algorithm verification.

Map	Evaluating Indicator	Baseline Group	This Paper	Variance
Town 01	Average vehicle speed	29.982 km/h	30.559 km/h	+1.92%
	Collision rate	0.9%	1.2%	+0.3%
	Destination arrival rate	99.1%	98.8%	−0.3%
	Emergency braking frequency	1.2518%	1.9713%	+0.7195%
	Control cycle	15.515FPS	14.522FPS	−0.993FPS
Town 02	Average vehicle speed	25.287 km/h	23.376 km/h	−7.56%
	Collision rate	1.1%	1.3%	+0.2%
	Destination arrival rate	98.9%	98.7%	−0.2%
	Emergency braking frequency	3.1342%	3.4576%	+0.3234%
	Control cycle	15.513FPS	13.497FPS	−2.016FPS
Town 03	Average vehicle speed	28.765 km/h	28.998 km/h	+0.81%
	Collision rate	0.8%	1.3%	+0.5%
	Destination arrival rate	99.2%	98.7%	−0.5%
	Emergency braking frequency	2.1036%	1.9853%	−0.1183%
	Control cycle	15.476FPS	17.632FPS	+2.156FPS
Town 04	Average vehicle speed	22.158 km/h	23.367 km/h	+5.46%
	Collision rate	0.9%	1.2%	+0.3%
	Destination arrival rate	99.1%	98.8%	−0.3%
	Emergency braking frequency	4.5269%	4.8732%	+0.3463%
	Control cycle	15.324FPS	17.983FPS	+2.659FPS

## Data Availability

Data are contained within the article.
